# A genetic screen identifies *Tor* as an interactor of VAPB in a *Drosophila* model of amyotrophic lateral sclerosis

**DOI:** 10.1242/bio.201410066

**Published:** 2014-10-31

**Authors:** Senthilkumar Deivasigamani, Hemant Kumar Verma, Ryu Ueda, Anuradha Ratnaparkhi, Girish S. Ratnaparkhi

**Affiliations:** 1 Indian Institute of Science Education and Research, Pune 411021, India; 2 National Institute of Genetics, Mishima, Shizuoka 411-8540, Japan; 3 Agharkar Research Institute, Pune 411004, India

**Keywords:** VAP, Neurodegeneration, TOR, ALS, *Drosophila* RNAi screen

## Abstract

Amyotrophic Lateral Sclerosis (ALS) is a progressive neurodegenerative disorder characterized by selective death of motor neurons. In 5–10% of the familial cases, the disease is inherited because of mutations. One such mutation, P56S, was identified in human VAPB that behaves in a dominant negative manner, sequestering wild type protein into cytoplasmic inclusions.

We have conducted a reverse genetic screen to identify interactors of *Drosophila* VAPB. We screened 2635 genes and identified 103 interactors, of which 45 were enhancers and 58 were suppressors of VAPB function. Interestingly, the screen identified known ALS loci – *TBPH, alsin2 and SOD1*. Also identified were genes involved in cellular energetics and homeostasis which were used to build a gene regulatory network of VAPB modifiers. One key modifier identified was *Tor*, whose knockdown reversed the large bouton phenotype associated with VAP(P58S) expression in neurons. A similar reversal was seen by over-expressing *Tuberous Sclerosis Complex* (*Tsc1,2*) that negatively regulates TOR signaling as also by reduction of S6K activity. In comparison, the small bouton phenotype associated with VAP(wt) expression was reversed with *Tsc1* knock down as well as *S6K-CA* expression. *Tor* therefore interacts with both *VAP(wt)* and *VAP(P58S)*, but in a contrasting manner. Reversal of VAP(P58S) bouton phenotypes in larvae fed with the TOR inhibitor Rapamycin suggests upregulation of TOR signaling in response to VAP(P58S) expression.

The VAPB network and further mechanistic understanding of interactions with key pathways, such as the TOR cassette, will pave the way for a better understanding of the mechanisms of onset and progression of motor neuron disease.

## INTRODUCTION

ALS is a late onset neurodegenerative disorder that leads to the dysfunction and death of motor neurons. While most cases of ALS are sporadic, 5–10% are known to be familial due to mutations in specific genetic loci (Pasinelli and Brown, 2006; Wijesekera and Leigh, 2009). Since the discovery of SOD1 (also called ALS1), over twenty different genetic loci have been shown to be associated with onset or progression of ALS (Andersen and Al-Chalabi, 2011). These loci include proteins with diverse functions and cellular locations, lacking a common thread connecting each of them to each other and to the disease. VAMP Associated Protein B (VAPB; ALS8) was first identified in *Aplysia* as an interactor of Vesicle Associated Membrane Protein (Skehel et al., 1995). A mutation in VAPB was later identified as the cause of familial ALS in a large Brazilian family (Nishimura et al., 2005; Nishimura et al., 2004). VAPB is a trans-membrane protein that is highly conserved from yeast to mammals (Lev et al., 2008; Nishimura et al., 2004). The protein contains an N-terminal MSP domain, a central coiled coil domain and a C-terminal trans-membrane domain (Nishimura et al., 1999; Skehel et al., 1995). A substitution mutation in the highly conserved proline residue at the 56^th^ position to Serine in the MSP domain results in a protein that forms cellular aggregates (Nishimura et al., 2004; Teuling et al., 2007). Patients with this mutation show spectrum of symptoms including typical ALS, slow progressive ALS and Spinal Muscular Atrophy (SMA) (Nishimura et al., 2004). This suggests that other genetic factors might decide the phenotypic outcome of the mutation.

VAP proteins (supplementary material Fig. S1A,B) perform an array of cellular functions: the MSP domain of VAPB interacts with proteins having FFAT motif to regulate lipid transport (De Vos et al., 2012; Lev et al., 2008; Peretti et al., 2008; Tsuda et al., 2008; Yang et al., 2012). The cleaved MSP domain acts as a ligand for Ephrin, Robo and Clr-1 receptors (Han et al., 2012; Tsuda et al., 2008); its interaction with Nir (N terminal interacting receptor) group of proteins modulates the ER and microtubule architecture (Amarilio et al., 2005; Skehel et al., 2000). VAPB also regulates Unfolded Protein Response (UPR) by interacting with components of two major UPR pathways, Ire1 and ATF6 (Gkogkas et al., 2008; Nikawa et al., 1995). The mutant form of VAPB (P56S in humans, P58S in *Drosophila*) is believed to cause the disease by acting in dominant negative manner, sequestering wild type protein into inclusions or by its failure to interact with other proteins (Mitne-Neto et al., 2007; Ratnaparkhi et al., 2008; Teuling et al., 2007; Tsuda et al., 2008).

In our study here, we have used reverse genetics to identify interactors of dVAP (henceforth referred to as VAP) – the *Drosophila* ortholog of hVAPB. Over-expression of VAP in sensory neurons using *scabrous-Gal4* (*Sca-Gal4*) leads to loss of thoracic bristles (Ratnaparkhi et al., 2008). Using this phenotype, we have screened approximately 17% of the fly genome to identify genes that interact with VAP. We have analyzed these interactions using the gene network approach and find that known ALS loci, *superoxide dismutase 1 (sod1)*, *vap, alsin and tar binding protein homolog (TBPH)* appear to be part of a connected regulatory network. A key finding is the identification of the Target of Rapamycin (TOR) pathway (Harris and Lawrence, 2003; Hoeffer and Klann, 2010; Johnson et al., 2013; Zoncu et al., 2011) as an interactor of VAP.

Using the larval neuromuscular junction (NMJ) as an assay system, we show that both *VAP(wt)* and *VAP(P58S)* interact with *Tor*, albeit in an opposing manner. In animals expressing VAP(P58S), down regulating TOR activity by either feeding animals Rapamycin, expressing a dominant-negative form of TOR, upregulation of *tuberous sclerosis complex* (TSC1, 2) activity, or decreasing S6 kinase (S6K) activity rescues the NMJ phenotype associated with VAP(P58S). Our results thus link expression of VAP(P58S) in neurons to modulation of TOR signalling – a well defined pathway involved in regulating nutrient sensing, cell growth and aging, suggesting that TOR mediated altered cell metabolism might contribute to VAP(P58S) mediated ALS.

## MATERIALS AND METHODS

### Fly strains, RNAi lines and genetic screen

Fly lines were maintained at 25°C on standard cornmeal agar medium. UAS-GAL4 system was used for over-expression of transgenes. All RNAi lines used in screen were from NIG, Japan. Given the large number of candidates involved, the efficacy of knockdown could not be determined for individual lines. However, many of these lines have been tested for functionality using an *actin-Gal4* driver. This information can be found at NIG-FLY website (http://www.shigen.nig.ac.jp/fly/nigfly/index.jsp).

*UAS-VAP* wild type and *UAS-VAP(P58S)* lines used in this study have been described earlier (Ratnaparkhi et al., 2008). Canton S flies were used as a wild type control. *UAS-Atg1* line was kindly provided by Dr. Chen, Academia Sinica. The following lines were obtained from Bloomington stock center: BL7013 (*TOR-DN*), BL6911 (*S6K-DN*), BL6914 (*S6K-CA*), BL33951 (*TOR*-TRIP RNAi), BL31314 (*Tsc1* RNAi), BL24854 (*Thor CA/ThorLL*).

### Primary and secondary screen

A recombinant fly line over-expressing wild type *VAP* using *sca* driver was used for the screening. For screening, males from RNAi stocks were crossed to virgins of *sca>VAP* over-expression line. The primary screening was performed at 25°C and 28°C. The secondary screen was performed only at 25°C. The Dorso-central, Scutellar and Anterior Postalar thoracic macro chaetae on F1 progeny females were considered for the analysis. The statistical significance was analyzed using Student's t-test.

### Data analysis

The Gene Ontology (GO) information was obtained based on Flybase (http://flybase.org) entries and GOToolbox (http://genome.crg.es/GOToolBox) online application and was grouped manually. For identifying the physical interactors of the modifiers we queried all the target genes against the GeneMANIA (http://www.genemania.org) and STRING (http://string-db.org) databases. To obtain all possible interaction we included low confidence interactions. Only known and predicted protein–protein interaction data based on other organisms was considered for identifying interactions. To identify physical interaction within genetic interaction, the information from these databases was curated manually. The network map was constructed using Cytoscape V2.8.3. Ortholog prediction was performed using DIOPT and DIOPT-DIST tools (http://www.flyrnai.org/diopt). Score of 1 on a scale of 10 was set as threshold to consider a gene as an ortholog. DIOPT combines information from different prediction tools to suggest a possible ortholog.

### Immunohistochemistry and imaging

For NMJ analysis, animals were raised in standard cornmeal agar medium at 28°C. Wandering third instar female larvae were dissected in cold PBS and fixed in Bouin's fixative for 20 minutes, except for p-S6K staining where larvae were fixed with 4% PFA in PBS. The tissues were washed, blocked in PBS containing 0.3% Triton X-100 and 2% BSA and incubated with primary antibody at 4°C overnight. Samples were washed and incubated with secondary antibody for overnight at 4°C. Then samples were washed and mounted using 70% glycerol with n-propyl gallate. The following antibodies were used for immuno-staining, Anti-HRP (1:500, Sigma) and anti-dlg (1:100, DSHB), anti-p-S6K (1:100, Cell signaling). Alex flour 488 and 568 secondary conjugate antibodies (1:1000) were obtained from Molecular Probes, Invitrogen. Synapse of muscle 4 in A2 and A3 segments were imaged for bouton size measurement. Only dlg positive type1b boutons were considered for analysis. The largest diameter of the every bouton in NMJ of muscle 4, excluding junctional boutons was measured. Confocal imaging was carried out using a Zeiss LSM 710 microscope. The bouton measurements were compared for significance using ANOVA.

### Rapamycin feeding

20 virgin females were allowed to mate for 24 hours and transferred to vials containing standard cornmeal media mixed with either DMSO or Rapamycin (200 nM, Invitrogen). Rapamycin at doses ranging from 200–1000 nM was tested, with 200 nM chosen as the dose for our experiments, based on increasing lethality at doses >800 nM. Larvae were continuously raised on Rapamycin/DMSO containing food and dissected at third larval instar stage for bouton analysis.

### Western blotting

10–15 third instar larval brains were collected and immediately lysed in Laemmli loading buffer (2×) with Phosphatase inhibitor cocktail (sigma) and Sodium Vanadate. Extracts were cleared by centrifugation and were run on 10% poly-acrylamide gel and transferred to PVDF membrane. Primary antibodies used were, Rabbit anti-p-S6K (1:1000, Cell Signaling), Rabbit p-4EBP1 (1:1000, Cell Signaling) and Mouse anti-tubulin DM1A (1:20,000, Sigma). HRP conjugated secondary antibodies were used at 1:10000 (Jackson Immunoresearch). Blots were developed using Immobilon luminal reagent (Millipore) using a LAS imaging system. The p-S6K levels were normalized to tubulin and quantitation was performed using ImageJ software (US-NIH).

## RESULTS

### A genetic screen uncovers interactors of VAP

**Fig. 1.**
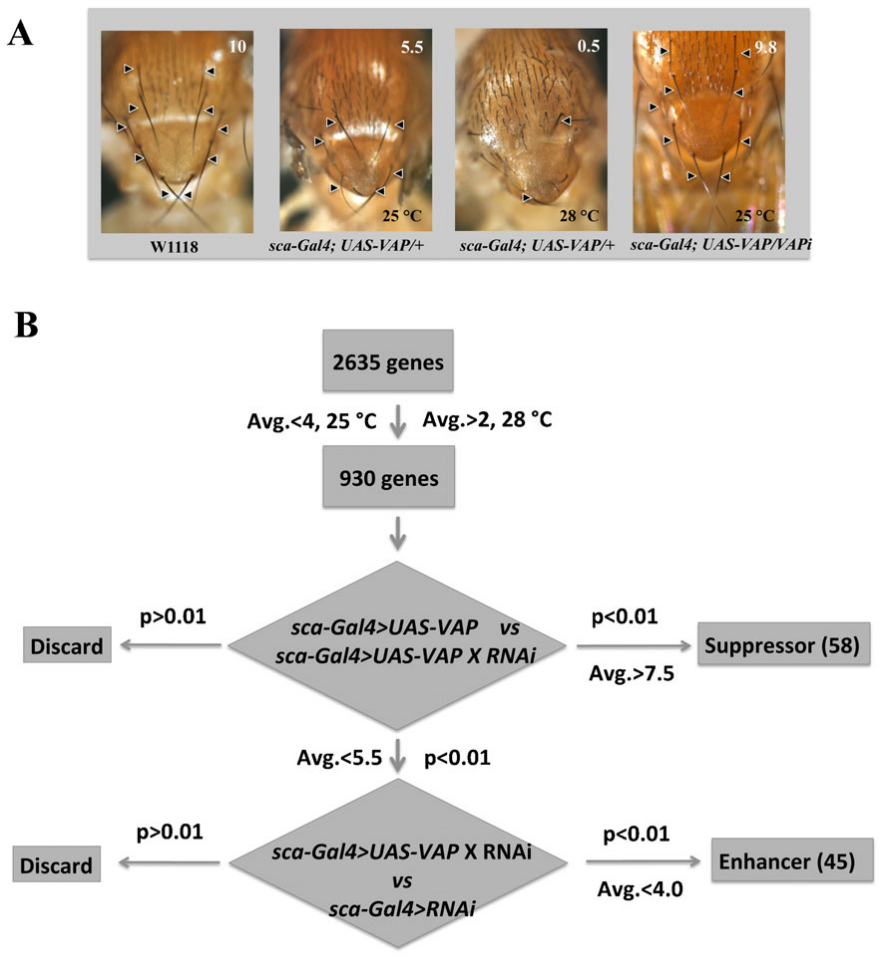
Scheme for the enhancer/suppressor screen. (A) A sensitized genetic background (*sca-Gal4,UAS-dVAP*) was used for a screen to identify interactors of dVAP. A recombinant stable line, expressing *VAP* in the *sca* domain was found to reduce the number of 10 macro chaetae (marked by arrowheads in *w1118*) to about 5–6 at 25°C. At an increased VAP dosage, at 28°C, the macro chaetae reduced to 0–1. Expressing dsRNA for *VAP*, in a dVAP over-expression background, led to a reversal of the phenotype with macro chaetae reverting to wild type levels both at 25°C and 28°C. Numbers at the top right hand corner of each picture are average macro chaetae, counted for ten females of the corresponding genotype. (B) Primary screening was done at both 25°C and 28°C. A *sca-Gal4, UAS-VAP/Cyo* recombinant, stable line was generated and females from that line were crossed to males with different transgenic RNAi inserts. Genes that lead to a further decrease of macro chaetae (from 5–6) at 25°C were deemed enhancers and genes that increased number of macro chaetae (from 0) at 28°C were considered to be suppressors. 2635 genes, encompassing 4600 individual lines were used for the primary screen. 930 genes showed change in the phenotype and were categorized as modifiers. 930 genes identified in the primary screen were used for a thorough, quantitative screening, with controls, at 25°C. Macrochaetae from ten F1 females were counted and compared to the base line 5.5 macro chaetae in the master control (*sca-Gal4, UAS-VAP/+*). Student's t-test was used to select lines that had significantly greater macro chaetae and these were considered *bona-fide* suppressors. Lines that did not meet our threshold for significance (p>0.01, Average macro chaetae <7.5) were discarded. F1 females with average macro chaetae <5.5 were compared to the related RNAi control (*sca-Gal4/+; UAS-RNAi/+*) at 25°C. Again, a Student's t-test was used to select lines above our threshold for significance (p<0.01, average macro chaetae <4). Starting with 2635 genes in the primary screen, the final numbers for enhancers and suppressors after comparison with controls and rigorous statistical analyses was 45 and 58 respectively. These genes were shortlisted for the validation process.

Stable expression of VAP in the *sca* domain leads to a dose dependent decrease in the number of macro chaetae in the dorsal thoracic region of the adult fly (Ratnaparkhi et al., 2008) (Fig. 1A). The penetrance of this phenotype is 100% with all the flies showing loss of bristles. At 25°C, the average number of macro chaetae observed in the stable recombinant lines (*Sca-Gal4>UAS-VAP/Cyo*) generated in our laboratory was between 5–6 macro chaetae and this number was seen to reduce further to 0–1 at 28°C. This phenotype was suppressed by co-expression of double stranded RNA (dsRNA) specific to VAP (VAP-RNAi, Fig. 1A). The loss of bristle phenotype was not significantly affected by the presence of an extra copy of UAS over-expressing RFP, eliminating the possibility of Gal4 dilution. The *Sca-Gal4>UAS-VAP/Cyo* is viable only as a heterozygote, and was used as a sensitized background to identify modifiers of VAP function through an RNAi based screen.

We utilized the publicly available RNAi collection (Kambris et al., 2006) (http://www.shigen.nig.ac.jp/fly/nigfly/about/aboutRnai.jsp) from National Institute of genetics (NIG, Japan) to screen for the modifiers. 4600 individual lines, representing 2635 genes were crossed to *Sca-Gal4>UAS-VAP* females and raised at 25°C and 28°C to screen for enhancers and suppressors respectively (Fig. 1B). 5–10 F1 females were screened from each cross; animals with <4 bristles at 25°C were scored as enhancers and those with >2 bristles at 28°C were scored as suppressors (Fig. 1B). Using these criteria, 930 genes were identified as modifiers of the phenotype. Of the 2635 genes screened, 2095 had orthologs in humans, based on DRSC Integrative Ortholog Prediction Tool (DIOPT) (Hu et al., 2011).

Next, in order to eliminate any effects of RNAi itself and to quantify the interaction, the 930 RNAi lines were rescreened at 25°C by crossing to *sca-Gal4* and *Sca-Gal4>UAS-VAP* (Fig. 1B). Bristle numbers of ten F1 females from both control and experimental crosses were counted and analyzed in two steps. In the first step, using 5.5 macro chaetae as the baseline (*sca-Gal4>UAS-VAP/+*), F1 females that showed significant rescue in macro chaetae number (average macro chaetae >7.5, p<0.01) were grouped as suppressors (Fig. 1B). Lines/genes with macro chaetae number 5.5–7.4 and p>0.01 were not considered significant hits. In the second step, control RNAi crosses for individual genes were taken into consideration. The effect of individual RNAi lines (*Sca-Gal4>gene*-i) on bristle number was compared to corresponding experimental value (*Sca-Gal4>UAS-VAP/gene*-i) in order to negate the effects of knockdown of the gene alone. Any line that did not have a strong effect on its own but lead to a further loss of macro chaetae in a VAP over-expression background was scored as an enhancer (average macro chaetae <4.0, p<0.01).

Using this strategy, we identified 45 enhancers and 58 suppressors of VAP phenotype (Fig. 1B; supplementary material Table S1). Of these, 89 genes (86%) have known human orthologs predicted using DIOPT (Hu et al., 2011). Using information from Flybase and DAVID (Huang et al., 2009; Marygold et al., 2013), we classified all modifiers into different categories (supplementary material Fig. S1D) based on their known or predicted functions. These included mitochondrial proteins, RNA binding proteins (*Arsenic resistance protein 2, CG7564* – a component of the spliceosome commitment complex), proteins associated with the cytoskeleton (*tropomodulin, dynactin subunit p-25, slingshot, zye*) and proteins associated with the Unfolded protein response (*Droj2, Hsp83, l(2)35Cc*). The largest class was a group of 10 genes, predicted to have roles in energy homeostasis or mitochondrial function (supplementary material Fig. S1F). For example, Prx5, a protein involved in redox homeostasis was identified as a strong suppressor while Tor was identified as a strong enhancer. Another interesting group of interacting proteins were those involved in nuclear import–export. Embargoed, a protein involved in nuclear export was identified as a strong enhancer in this while other nuclear import–export components like *Nup75*, *CG8219* and *karyopherin-β3* were also picked up as enhancers.

### A genetic network for dVAP

**Fig. 2.**
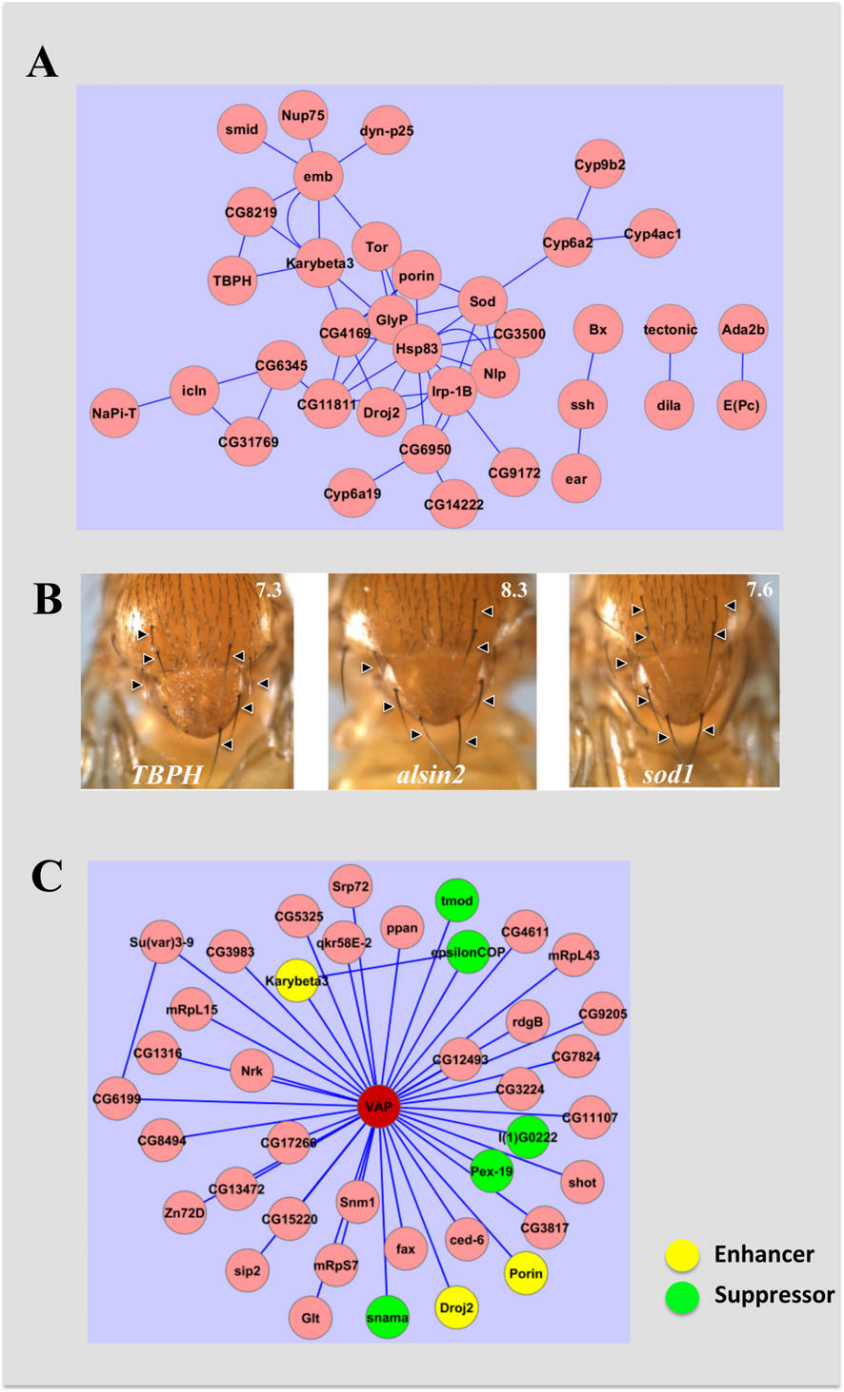
An integrated network of dVAP genetic interactors. An extended network of dVAP network was built by integrating VAP interaction data from our screen (103 genes) with known physical interactors of dVAP. The extended network (not shown) includes 406 genes and 953 edges. (A) Analysis indicated that 36 modifiers (displayed as circles), which are a subset of the 103 modifiers discovered, interact physically among themselves. Blue connecting lines (edges) indicate physical interaction. (B) *Drosophila* homologs of known ALS causing loci, *TBPH*, *Alsin* and *sod1* are suppressors of dVAP function and are part of the genetic network. The genotype for each cross is *Sca-Gal4/+; UAS-VAP/UAS-Gene-RNAi*. For each figure, average macro chaetae values from ten females are represented on the top right hand corner. (C) Thirty-five known physical interactors of VAP are a subset of the 2635 genes screened in this study. Of these, eight genes were found to be *genetic* interactors of VAP. *SNAMA, tmod, lethal (1) G0222, epsilon-COP* and *Pex-19* are suppressors while *Droj2, karyopherin beta3, Porin* are enhancers.

In order to identify components of the extended VAP network we built an interaction network between the modifiers identified in our screen and physical interactors of VAP using interactions from the databases STRING and GeneMANIA (Franceschini et al., 2013; Mostafavi et al., 2008) for *Drosophila* proteins. We considered only direct interactions and those separated by single node for building the integrated network. Our extended network contains 406 proteins, connected by 953 edges. Of the 103 modifiers identified in our screen, we found that 36 (35%) physically interact among themselves, connected by 53 edges (Fig. 2A). Of these, 13 physical interactions involving 12 proteins are ranked as medium confidence interactions (Combined score or weight of ≧0.4) by STRING or GeneMANIA. We also found that 61 (59%) of the genetic interactors can be linked via a common physical interactor. Based on predictions by DIOPT-DIST and literature mining, 23 of the 103 genes (22%) identified have been implicated to be involved in or regulating a human nervous system disease (supplementary material Table S2). This is significant considering that there is increasing evidence pointing to the idea of common network of genes/processes that are affected in several neurodegenerative diseases (Chen and Burgoyne, 2012; Shulman and De Jager, 2009). Our analysis leads us to believe that we have identified a core network of genes and proteins that interact with VAP and thus have the ability to modulate and be modulated by VAP function.

### Known ALS loci and physical interactors of VAP act as modifiers

Recent studies have shown hVAPB levels to be affected in patients with sporadic ALS. SOD1 transgenic mice and VAP mutant mice show TDP-43 pathology suggesting that many of these pathogenic pathways may be linked. We therefore sought to see if any of the loci involved in ALS act as modifiers of VAP phenotype. Indeed, knockdown of *SOD1*, *Alsin2* and TBPH suppressed the loss of bristle phenotype (Fig. 2B) in *sca-Gal4>VAP* animals. The above results support the idea that causative genetic loci for ALS interact genetically with each other.

A subset of RNAi lines in our primary screen represented genes whose protein products are known to physically interact with VAP. Of the known thirty-five physical interactors that were tested in our screen, a significant fraction (8 genes; 22%), were genetic modifiers in our screen (Fig. 2C). Of these *SNAMA, Tropomodulin, lethal (1) G0222, epsilon-COP* and *Pex-19 (CG5325)*, were identified as suppressors and *Droj-2, Karyopherin-β3, porin* as enhancers. A physical interaction with a protein may or may not manifest in the phenotype being used to score for genetic interactions. Moreover, many of these interactions have been identified in S2 cells (Guruharsha et al., 2011) or tissues other than neurons. It is possible that some of these interactions are not represented in neurons, which is our tissue of interest.

### Modifiers identified in screen alter VAP(P58S) induced bouton size

The genetic screen was conducted to identify interactors of wildtype VAP. In order to evaluate the interaction of these genes with disease causing VAP(P58S), we used the *Drosophila* larval NMJ as an assay system. VAP regulates bouton size at the NMJ in a dose dependent manner (Pennetta et al., 2002; Ratnaparkhi et al., 2008). Over-expression of VAP(P58S) in neurons using pan-neuronal *C155-Gal4* driver, leads to enlarged boutons at the larval NMJ (Ratnaparkhi et al., 2008). To test interaction with VAP(P58S), we decided to knockdown a small subset (∼14%) of discovered interactors in the background of neuronal VAP(P58S) expression and test if the reduction in transcripts of individual interactors, identified in a VAP(wt) screen modified the enlarged bouton phenotypes. Fourteen of the 103 interactors were knocked down using RNAi in *CI55-Gal4>UAS-VAP(P58S)* animals. The choice of interactors was made so as to include suppressors (*CG18110, CG6048, NapiT, Prx5 and TBPH*), enhancers (*Ada2b, Ars2, CG9172, Droj2, Karβ3, Nup75, ssh* and *tor*) and known physical interactors of VAP (*Droj2, Karyβ3 and Snama*) that were identified as interactors in the genetic screen. Some of the interactors we chose also have roles in human disease (supplementary material Table S2).

**Fig. 3.**
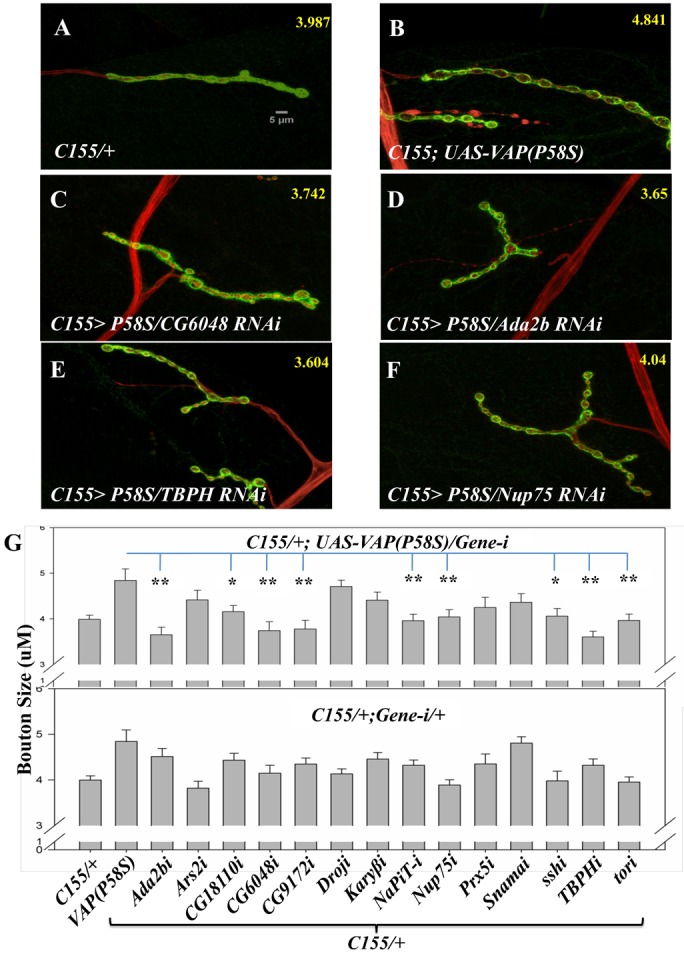
The *Drosophila* NMJ is used to screen for interaction of modifiers with VAP(P58S). Thirteen of the 103 modifiers discovered in our macro chaetae screen were tested in the larval muscle-4 NMJ for interaction with VAP(P58S). For this and subsequent figures, approximately 15 NMJs were dissected, stained (anti-HRP, red), imaged and measured for the average size of boutons (displayed in yellow at the top RHS of each figure). (A) A wild type (*C155-Gal4/+*) NMJ. The average bouton size is 3.987 (±0.03). Shown here and below is Z-series of a synapse rendered as maximum intensity projection. (B) Expression of *UAS-VAP(P58S)*, using the *C155-Gal4* driver increases the size of the boutons. (C–F) Knockdown of *CG6048* (C), *CG9172* (D), *TBPH* (E) *and Nup75* (F) in a *C155/UAS-VAP(P58S)* background reverses the effect of the VAP(P58S) over-expression and rescues bouton size to wild-type levels. (G) Quantitation of bouton size (in micrometer) for the RNAi knockdown of each gene tested in a VAP(P58S) background (top panel) and in a wild type background (bottom panel). For this and subsequent NMJ figures, error bars represent standard errors of the mean (SEM). Scale bar: 5 µm. * indicates a p-value<0.01 (but >0.001), while ** indicates a p-value of <0.001.

In control *C155-Gal4/+* animals, the average bouton size was 3.98±0.09 µm (n = 17); in *C155-Gal4>VAP(P58S)* animals, boutons are large with an average size of 4.84±0.25 µm (n = 16) (Fig. 3A,B respectively, p-value = 0.0016) without any significant change in the bouton number. Knockdown of *Ada2b, CG18110, CG6048, CG9172, NaPi-T, Nup75, Ssh, TBPH* and *Tor* suppressed the bouton phenotype observed in *C155-Gal4>VAP(P58S)* animals (Fig. 3C–F) while *Ars2, Droj2, Karyβ-3, Prx5, Snama* knock down failed to rescue or worsen the bouton size. We have interpreted the rescue of bouton size as a scaling back of the perturbation effect of VAP(P58S) on the pan neuron–glia–muscle network. Rescue of the bouton size did not seem to be additive since knockdown of these genes on their own (Fig. 3G, bottom panel), did not give rise to smaller boutons. On the contrary, the boutons were found to be marginally bigger as compared to the controls though many of them were not statistically significant except in the case of *Snama* and *Karyβ-3*, with bouton size increasing to 4.45±0.13 µm and 4.80±0.14 µm respectively (Fig. 3G). None of the genes we tested, by themselves, showed any significant increase in bouton size compared to *C155-Gal4>VAP(P58S)*.

### Modulation of TOR pathway components suppresses VAP(P58S) bouton phenotypes

**Fig. 4.**
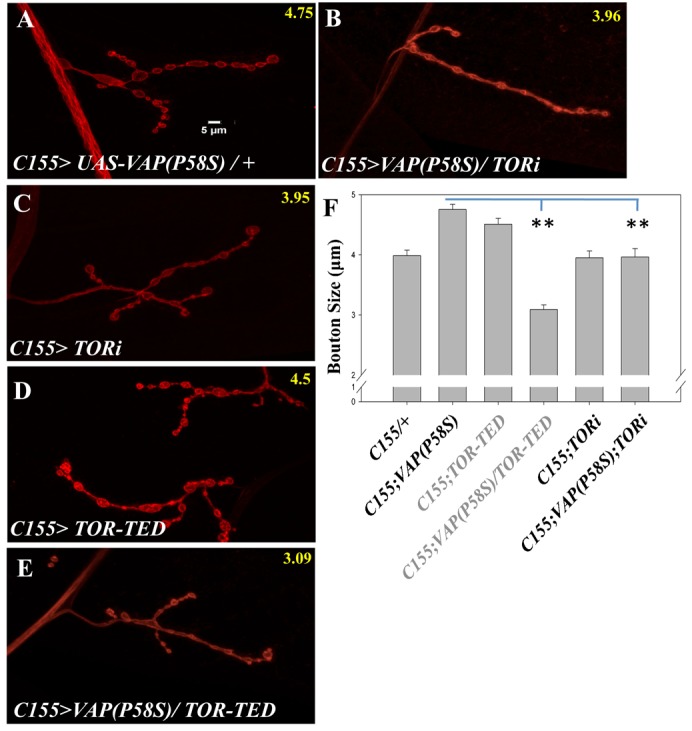
dTOR, the fly homolog of mTOR modifies the VAP(P58S) over-expression phenotype at the NMJ. (A) VAP(P58S) over-expression using *C155-Gal4* leads to larger boutons at the NMJ. (B) Knockdown of *Tor* by RNAi in VAP(P58S) background restores the bouton size to wild type levels. (C) Knocking down *Tor* using RNAi did not have any effect on bouton size. (D) Over-expression of *TOR-TED* alone using *C155-Gal4* resulted in increased bouton size. (E) Reduction of TOR activity by expressing a dominant negative form of TOR, *TOR-TED* reversed the increase in bouton size caused by *UAS-VAP(P58S)*. Scale bar: 5 µm. (F) Quantitation of rescue in bouton size in VAP(P58S) background in response to reduced TOR.

One of the enhancers we identified in the bristle-based screen was *Tor*. Knockdown of *Tor* suppressed the large bouton phenotype of VAP(P58S) (Fig. 4A,B), to wild type levels (4.75±0.08 µm to 3.96±0.09 µm, p-value = 0.0001). Knockdown of *Tor* by itself did not change the bouton size in comparison to *C155-Gal4* (3.98±0.03 µm vs 3.95±0.03 µm, p-value = 0.8115, Fig. 4C). This suggests that *VAP(P58S)* interacts with *Tor* and that these neurons may have increased TOR signaling.

To confirm this result, we tested if the dominant negative form of *TOR* (*TOR-TED*) affects NMJ phenotype in the *C155>VAP(P58S)* background. We found that TOR-TED when expressed in P58S background reduced the bouton size significantly compared to P58S over-expression (4.75±0.08 µm vs 3.09±0.07 µm, p-value = 0.00001, Fig. 4E). The decrease in bouton size was also significantly lower than the *Gal4* control (3.98±0.09 µm vs 3.09±0.07 µm, p-value = 0.001). In contrast, expressing only TOR-TED using *C155-Gal4* alone increased bouton size marginally (3.98±0.09 µm vs 4.50±0.10 µm, p-value = 0.0005, Fig. 4D).

**Fig. 5.**
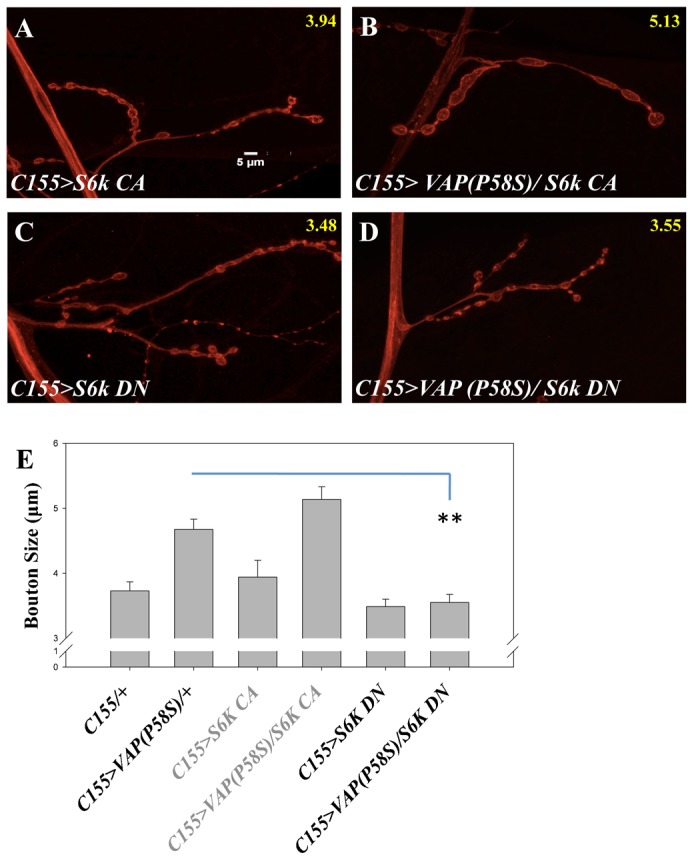
Increased TOR signaling activates its downstream component S6K. (A) Over-expression of constitutively active S6K using *C155-Gal4* in a wild type background did not affect the bouton size. (B) Over-expression of constitutively active S6K using *C155-Gal4* in a VAP(P58S) over-expression background did not reduce the bouton size. (C) Reducing S6K activity using dominant negative form did not have significant effect on bouton size. (D) Reducing S6K activity using dominant negative form rescued the increased bouton size in a VAP(P58S) background. Average size of boutons from about 15 NMJs is displayed in yellow at the top right of each figure. Scale bar: 5 µm. (E) Quantitation of effect of S6K on the bouton size in presence and absence of VAP(P58S).

In order to further validate the genetic interaction between VAP and TOR we tested downstream components (Fig. 5), namely S6K, *Autophagy* 1(*Atg1*) and *Thor* (*4EBP1*) of the TOR signaling cassette. TOR activates S6K by phosphorylating it and this promotes protein translation. Co-expressing constitutively active (CA) form of S6K (*UAS-S6K^STDETE^*) with VAP(P58S) did not rescue the bouton size (Fig. 5A,B), though there was a marginal increase that was not statistically significant (4.67±0.15 µm vs 5.13±0.19 µm, p-value = 0.0848, Fig. 5B). Over-expressing *S6K^STDETE^* with *C155-Gal4* did not result in any change in bouton size consistent with earlier results (3.72±0.14 µm vs 3.94±0.25 µm, p-value = 0.4651, Fig. 5A) (Cheng et al., 2011). However, when we co-expressed kinase-dead/dominant negative form (DN) of S6K using *UAS-S6K^KQ^* along with P58S form of VAP we observed a significant decrease in the bouton size (4.67±0.15 µm vs 3.55±0.12 µm, p-value = 0.0001, Fig. 5D). By itself, reduction of S6K activity via *UAS-S6K^KQ^* did not significantly decrease bouton size (3.72±0.14 µm vs 3.48±0.11 µm, p-value = 0.3654, Fig. 5C). In comparison, *S6K* null mutants show decreased bouton size without affecting the bouton number (Cheng et al., 2011). Co-expressing constitutively active *Thor* (*Thor CA*) form with VAP(P58S) did not rescue the large bouton phenotype (supplementary material Fig. S2D–F). Over-expression of *Atg1* in VAP(P58S) background leads to a decrease in bouton size (supplementary material Fig. S2B,C,F), a result negated by the control experiment (*C155>Atg1*).

**Fig. 6.**
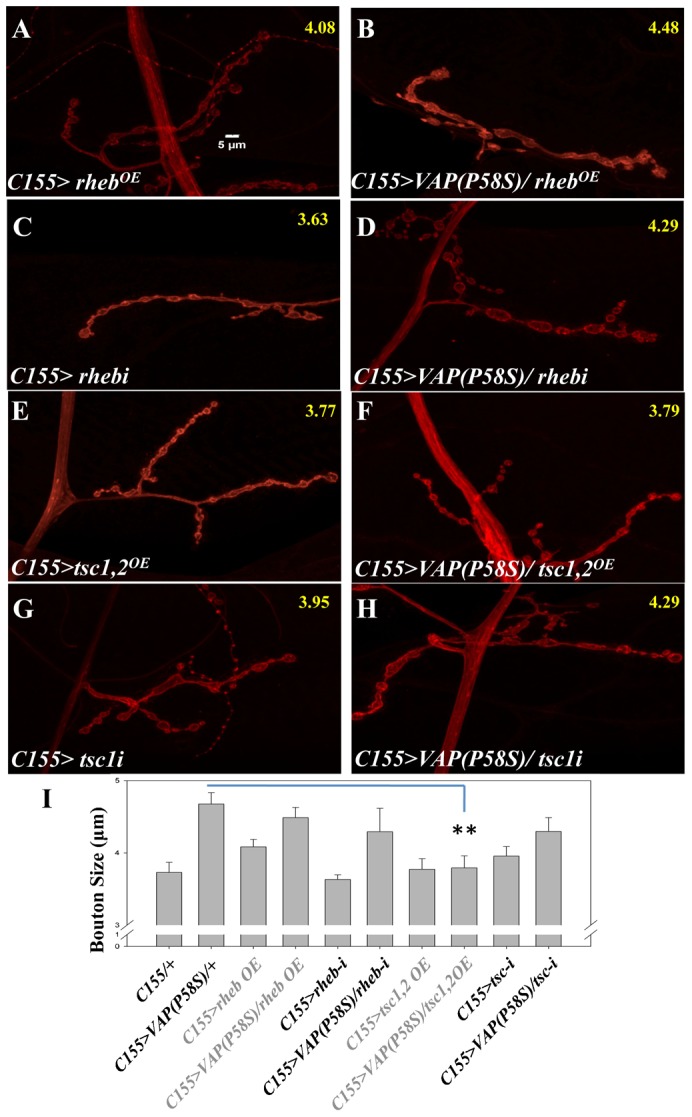
Increased TSC activity reverses the VAP(P58S) bouton phenotype. (A–D) Over-expression and knocking down of *Rheb* had no effect on bouton size in the VAP(P58S) background. (E,F) Increasing Tsc activity by co-expressing *Tsc1,2* rescued the bouton size in VAP(P58S) background (F) while *Tsc1,2* over-expression by itself did not have any effect on bouton size (E). (G,H) Knocking down *Tsc1* in VAP(P58S) background did not rescue the bouton size. Scale bar: 5 µm. Average size of boutons from about 15 NMJs is displayed in yellow at the top right of each figure. (I) Quantitation of effect of perturbations in *Tsc* and *Rheb* levels on bouton size.

The above results indicate that TOR may be upregulated in VAP(P58S) mutants. Knocking-down or over-expressing *Rheb* in VAP(P58S) background did not affect the bouton size (Fig. 6A–D) but co-expression of *Tsc1 and Tsc2*, rescued the bouton size back to wild type levels (4.67±0.15 µm vs 3.79±0.16 µm, p-value = 0.00067, Fig. 6E,F). As expected, knock down of *Tsc1* in VAP(P58S) background did not alter the bouton size (4.67±0.15 µm vs 4.29±0.19 µm p-value = 0.1621, Fig. 6G,H).

### Modulation of *Tor* pathway components suppresses VAP(wt) bouton phenotypes

Since *Tor* interacts with *VAP(P58S)*, we conducted equivalent experiments in a *VAP(wt)* over-expression (*C155>VAP*) background in order to compare and contrast the genetic interactions of *VAP(P58S)* and *VAP(wt)* with *Tor*.

**Fig. 7.**
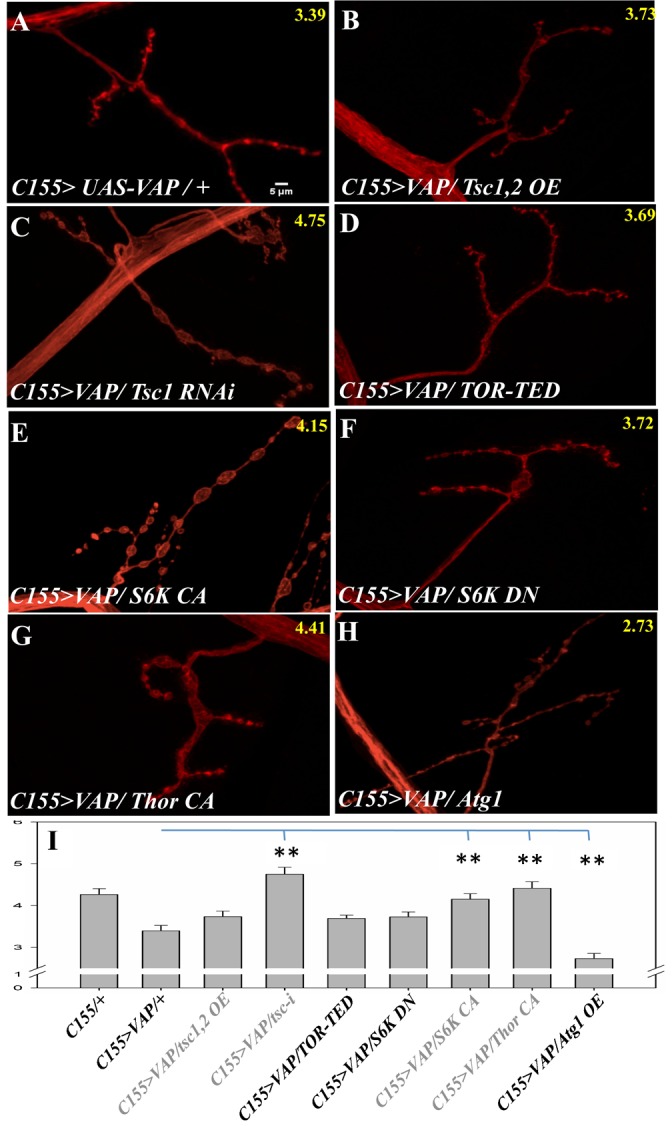
Decreased TSC activity and increased S6K activity reverses the VAP(wt) small bouton phenotype. (A) VAP wild type over-expression using *C155-Gal4* leads to reduced bouton size. (B–D) Reducing TOR pathway activity by over-expressing *Tsc1,2* (B) or *TOR* dominant negative (D) does not affect the bouton size in the VAP wild type over-expression background. However, increasing TOR pathway activity using *Tsc1* knock down increased the bouton size to wild types levels (C). (E–H) TOR downstream components can alter the bouton size in VAP wild type over-expression. Over-expression of a constitutively active form of S6K (E) and constitutively active Thor (G) rescues the bouton size, while the dominant negative form does not (F). Over-expression of Atg1 along VAP wild type reduced the bouton size further (H). Scale bar: 5 µm. Average size of boutons from about 15 NMJs is displayed in yellow at the top right of each figure. (I) Quantitation of effect of TOR pathway and its downstream components in VAP wild type over-expression mediated bouton size. Error bars represent SEM. * indicates a p-value<0.01 (but >0.001), while ** indicates a p-value of <0.001.

In a *VAP(wt)* background co-expressing *Tsc1,2* in did not rescue the small bouton phenotype (Fig. 7A,B,I). But down-regulation of *Tsc1* rescued the phenotype dramatically (3.39±0.13 vs 4.75±0.16, p-value<0.0001, Fig. 7A,C,I) suggesting lowered TOR signaling when VAP(wt) is expressed in neurons. *TOR-TED*, when expressed, did not modify the small bouton size (3.69±0.08 µm vs 3.39±0.13 µm, p-value = 0.055; Fig. 7A,D,I). *Thor CA*, when expressed in a VAP(wt) background, rescues the small bouton phenotype (3.39±0.12 vs 4.41±0.15 µm, p-value = 0.0002, Fig. 7G,I); but Thor CA by itself lead to larger boutons (supplementary material Fig. S3E). Co-expresssion of *Atg1* with *VAP(wt)* leads to smaller boutons, a phenotype negated by the fact that *Atg1* over-expression by itself leads to small boutons. *S6K-DN* in a *VAP(wt)* expression background does not change the small bouton phenotype but *S6K-CA* expression rescues the phenotype (3.39±0.12 µm vs 4.15±0.13 µm, p-value = 0.0003, Fig. 7E,I). This suggests that TOR signaling may be down regulated in neurons over-expressing VAP(wt).

### Rapamycin feeding mitigates VAP(P58S) phenotype

The above genetic data suggests an up regulation of TOR signaling in neurons expressing VAP(P58S). Based on this, one would predict that treatment with Rapamycin, which inhibits TOR signaling could reverse the large bouton morphology of VAP(P58S) expressing neurons. In addition, kinases downstream of the TOR pathway may show an increase in phosphorylation.

**Fig. 8.**
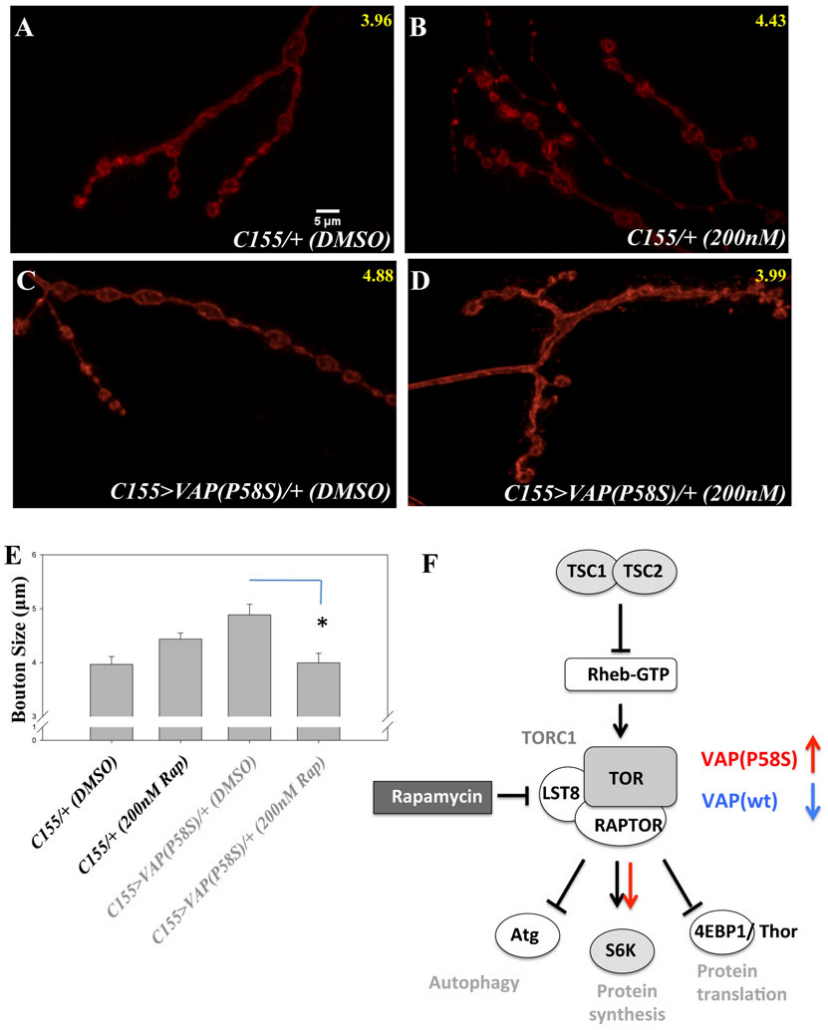
Rapamycin feeding mitigates VAP(P58S) bouton phenotype. (A,B) *C155-Gal4* larvae fed with DMSO (A) and 200 nM Rapamycin (B). (C,D) *C155>VAP(P58S)* larvae fed with DMSO (C) and 200 nM Rapamycin (D). Rapamycin, a chemical inhibitor of TOR effectively restores bouton size to control levels in VAP(P58S) animals. (E) Quantitation of effects of Rapamycin on VAP(P58S) expressing animals. (F) A model for the effect of VAP(P58S) expression on TOR. Genetic interactors (grey filled circles or rounded squares) of *VAP(P58S)* suggests an up-regulation of *Tor* (red arrows) in neurons while VAP(wt) expression appears to have opposite effects (blue arrow). Rapamycin feeding of larvae reverses VAP(P58S) phenotypes, pointing to an increase in TOR signaling in VAP(P58S) expressing neurons.

200 nM Rapamycin was fed to larvae of the appropriate genotype (C155-Gal4/+ and C155-Gal4/+; UAS-VAP(P58S)). A dose dependent study for Rapamycin was carried out (see Materials and Methods) to determine the correct dosage. Control *C155-Gal4* larvae showed a moderate increase in bouton size compared with their respective control (3.96±0.14 µm vs 4.43±0.11 µm, p-value = 0.0156, Fig. 8A,B). VAP(P58S) expressing larvae, when fed with Rapamycin, showed a significant decrease in bouton size, compared to DMSO fed controls (4.88±0.19 µm vs 3.99±0.17 µm, p-value = 0.0021, Fig. 8C,D), confirming that inhibiting TOR pathway can reverse the VAP(P58S) mediated bouton phenotype. We also tried to measure the levels of p-S6K in larval brains lysates using western blotting using an antibody raised against mammalian p-S6K and 4EBP1. However, we failed to detect any significant change in the levels of p-S6K with western blots, carried out using brain lysates from larvae expressing VAP(P58S), based on four different biological replicates (supplementary material Fig. S3). Thus, inhibiting TOR signaling by feeding larvae Rapamycin appears to be sufficient to reverse VAP(P58S) bouton phenotypes.

## DISCUSSION

### A genetic screen that enriches VAP modifiers

VAP was originally identified as an interactor of VAMP involved in vesicle release (Skehel et al., 1995). In the last few years many mutations in VAP leading to ALS have been identified (Chen et al., 2010; Han et al., 2013a; Kabashi et al., 2013; van Blitterswijk et al., 2012). It has also been observed that levels of VAP increase in case of some tumors (Rao et al., 2012). Recent studies have identified a number of roles for VAP and how these functions might be affected in case of a mutation (Chen et al., 2010; Han et al., 2013a; Kabashi et al., 2013; van Blitterswijk et al., 2012).

We report the first screen to identify modifiers of ALS8 gene VAP using a *Drosophila* over-expression model. The outcome of the screen, which is a list of genetic interactors, is evaluated with two primary concerns; one regarding the modifiers being related to a general cellular phenomenon such as proteostasis rather than VAP function and the second, the possibility of identifying large number of interactors, indicating false positives. In order to negate the first possibility we compared our modifiers with genes involved in protein homeostasis (Silva et al., 2011) as well as the ERAD network (Christianson et al., 2012). None of the modifiers from our screen were a part of the nine genes identified as regulators of proteostasis network and although thirteen of the ERAD network proteins were represented in our screen, we identified only *Hsp83* as in interactor. The second concern was related to the high number of interactors identified, which was about 3.9% of the total genes screened. This would, in a normal scenario, indicate that our screen is identifying non-specific genetic interactions. This finding has to be balanced by the fact that VAP has been linked a large repertoire of functions within and outside the cell. In a genome wide screen performed by radiation hybrid method in cell lines, larger numbers, namely 2500 genetic interactors of VAP and VAPA have been identified. 19 of the 103 (18.4%) interactors in our screen have been identified in this and other genome wide screens in other organisms (Costanzo et al., 2010; Lin et al., 2010) (supplementary material Table S3). One hit in our screen is *Tor*, which itself interacts with many housekeeping and homeostatic cellular components, leading to a possible increase in hits in our screen.

### VAP modifiers and ALS

The discovery of *TBPH*, *alsin2* and *sod1* as modifiers in our screen implies that even in a wild type scenario, these genes are part of the VAP genetic network. There is increasing evidence to suggest that interplay exists between different ALS causing loci and that ALS causing loci may be part of a core network involved in determining the progression of neuro-degeneration. Further evidence for this thesis come from earlier data that indicates that VAP(P58S) mutation affects localization of sigma receptor 1 (Prause et al., 2013), another ALS locus. Localization of TDP-43 – another gene associated with ALS, is also affected because of the P58S mutation (Tudor et al., 2010). Supporting this, we show that loss of *Drosophila* TDP-43 is able to alter the VAP(P58S) mediated bouton size (Fig. 3E,G).

Additional evidence for interactions between VAP and other ALS loci comes from the finding that VAP levels are lowered in sporadic ALS patients and mice expressing mutant SOD1 (Teuling et al., 2007). A recent study in *smn* based spinal muscular atrophy (SMA) shows that *SOD1* can alter the bouton morphology caused because of *smn* knock down (Sen et al., 2013). Our screen identifies *SOD1* as an interactor of VAP. Our observation together with other studies implies that many if not all ALS causing loci interact with each other and a core network exists in cells, which is sensitive to perturbation of its individual members and has a tendency to breakdown in motor neurons late in life.

### VAP and cellular homeostasis

A GO analysis of the 103 modifiers as well as their human counterparts identifies clusters of genes related to protein trafficking, lipid biosynthesis, protein biogenesis, stress and cellular energetics. These categories include some of the possible cellular functions that researchers increasingly believe to be affected in neurodegenerative diseases (supplementary material Table S2) in general and ALS in particular. The role of VAP in lipid biosynthesis is best understood with its association with ceramide transfer protein (CERT) (Peretti et al., 2008; Perry and Ridgway, 2006). In our screen, we identify novel genes implicated in lipid and inositol metabolism, namely CG33090, CG14222 and CG9391 whose characterization may lead to a better understanding of VAP function. Secreted MSP domain of VAP regulates mitochondrial morphology (Han et al., 2012) and VAP(P56S) mutant is known to affect anterograde transport of mitochondria along the axons (Mórotz et al., 2012). VAP interacts with PTPIP51 and helps in maintaining calcium homeostasis (De Vos et al., 2012). Neuronal loss of VAPB affects reduces ATP levels, altered fat metabolism in muscles and this is mediated via DAF-16 (Han et al., 2013b). In our screen, *Bmcp, CG4169, CG3476, CG9172, porin, Prx5* and *mRpS34* (supplementary material Fig. S1F) are mitochondrial proteins that may further link VAP to mitochondrial function. VAP misfolding and aggregation causes ER stress (Gkogkas et al., 2008; Moustaqim-Barrette et al., 2014), a phenomenon that may involve *Droj*, *Hsp83* and *l(2)35Cc*. We also identified nucleoporins as interactors of VAP. siRNA mediated knock down of VAP results in cytoplasmic retention of Nup-214 and emerin; and VAP (56S) mutant leads to nuclear envelope defects (Tran et al., 2012). TOR, which is the focus of our study, is a major player in cellular homeostasis and disease (Laplante and Sabatini, 2012). Thus, many of the genes identified in our screen, though novel, fall in the same categories of known VAP functions and an understanding of their function in relation to VAP and VAP(P58S) may lead to a better understanding of the disease mechanisms.

### *VAP(P58S)* and *VAP(wt)* over-expression indicates differential interactions with *Tor*

Mammalian TOR (mTOR), an atypical serine–threonine kinase is an anabolic promoter activated by insulin receptor that increases protein synthesis while inhibiting autophagy (Fig. 8F). In humans TOR signaling appears to occur via two independent complexes, TORC1 and TORC2. The TOR complexes along with FKBP12 were identified in yeast as targets of the fungal immunosuppressant drug Rapamycin (Heitman et al., 1991). mTOR accepts signals, integrates the information and regulates downstream cellular functions such as autophagy, cytoskeleton rearrangements and protein synthesis (Johnson et al., 2013). S6K and TSC complex have recently been show to play a role in regulating *Drosophila* NMJ development (Cheng et al., 2011; Natarajan et al., 2013), affecting bouton size and number while knockdown of *Tor* does not appear to modulate bouton number (Penney et al., 2012).

Reducing TOR activity by either RNAi interference, expressing Dominant negative (DN) constructs or by reducing activity of its downstream effector S6K, rescued the bouton size in VAP(P58S) background suggesting that the TOR pathway may be upregulated by VAP(P58S). Over-expression of TSC complex components as well as feeding larvae Rapamycin rescued the bouton size. The effect of Rapamycin is consistent with its historical role as a TOR signaling inhibitor.

Expression of a constitutively active form of *Thor* did not modulate the effects of VAP(P58S) expression, and we could not elucidate the effects on Atg1 activity because it had a strong effect on bouton size in control experiments. In addition to biochemical experiments that related to inhibiting TOR signaling using Rapamycin, we also attempted to visualize changes in Phosphorylation for S6K and Thor by measuring levels of phosphorylated antibody within the cell in conditions of VAP(P58S) over-expression (see Results).We could not detect any increase in phosphorylation and this may be because of technical limitations of our experiment – namely that a large background of p-S6K from other cells may not allow detection of the change in phosphorylated states; the antibody used is a mammalian p-S6K antibody that cross-reacts with *Drosophila* p-S6K, and an antibody that recognizes total S6K is not available.

In a background of VAP(wt) over-expression, TOR signaling appears to be decreased, in contrast to its likely upregulation in VAP(P58S) background. The strongest evidence for this is the rescue of the small bouton phenotype of VAP(wt) when *tsc1* levels are reduced or when S6K-CA are expressed in the same neurons.

### Rapamycin, TOR inhibition and human neurodegenerative disease

Upregulation of TOR signaling appears to be a common feature of the progression of neurodegenerative diseases such as Alzheimer's, Parkinson's and Huntington's, in animal models, with inhibition of signaling by rapamycin reversing cognitive and motor deficits (Ravikumar et al., 2004; Spilman et al., 2010), attenuating development of posttraumatic epilepsy, reducing aggregation (Caccamo et al., 2010), promoting autophagy (Floto et al., 2007; Ravikumar et al., 2004) and protecting against neuronal cell death (Malagelada et al., 2010; Wang et al., 2009).

TOR pathway has been shown to be involved regulating aging and a number of diseases. Reduced TOR signaling has been shown in case of VCP mediated ALS (Ching et al., 2013). Feeding SOD1^G93A^ mutant mice with rapamycin, an inhibitor of TORC1 shortens life span (Zhang et al., 2011). Treating N2A cells expressing mutant *TDP-43* with rapamycin has been shown to alter the localization of protein (Caccamo et al., 2009). Increased TOR activity has been observed in case of Fragile X syndrome, Huntington's, PINK1 based Parkinson's disease models (Liu and Lu, 2010). Increased TOR activity (Figs 6–8) may lead to reduced autophagy or increased global translation in these disease models. Deregulated protein translation is believed to cause energy imbalance in the cell and lead to tissue degeneration. Supporting this idea, it has been shown that ATF4 and CHOP, the two ER stress responsive transcription factors upregulate protein synthesis genes (Han et al., 2013a). The increased protein translation results in depletion of cellular ATP and induces ROS production, which eventually activates apoptotic pathways in cell. Interestingly, recent studied in VAP (P56S) transgenic mice models show increased nuclear levels of ATF4 and CHOP (Aliaga et al., 2013). In *apoe/ldlr* based atherosclerotic model and Po glycoprotein based Marie–Charcot Tooth disease model it has been observed that reducing ATF4 and CHOP activity mitigates the disease pathogenesis (Pennuto et al., 2008; Thorp et al., 2009). Pharmacological interventions targeting components of the TOR pathway might help in alleviating the progression of ALS.

In summary, our genetic screen uncovers a genetic network for *Drosophila* VAPB. A similar genetic network should exist in humans. We demonstrate that some members of the network can reverse NMJ phenotypes of VAP(P58S) expression in neurons, indicating that these modifiers are possible targets to understand the mechanism of VAP mediated ALS as well as potential drug targets. *Tor*, an important regulator of cellular function interacts differentially with both *VAP(P58S)* and *VAP(wt)*. The TOR inhibitor rapamycin reverses the architectural effects on VAP(P58S) expression on the *Drosophila* NMJ. A more detailed study of the *Tor*-*VAP* interaction should provide insight into the mechanism of progression of ALS8.

## Supplementary Material

Supplementary Material
