# Supplementary material for: A genetic screen identifies Tor as an interactor of VAPB in a Drosophila model of amyotrophic lateral sclerosis
Source: Biol Open. 2014 Oct 31;3(11):1127–38. doi: 10.1242/bio.201410066 (PMC4232771; doi:10.1242/bio.201410066)
Supplement: Supplementary Material [file supp_3_11_1127__index.html]

A genetic screen identifies Tor as an interactor of VAPB in a Drosophila model of amyotrophic lateral sclerosis — Supplementary Material 

# A genetic screen identifies *Tor* as an interactor of VAPB in a *Drosophila* model of amyotrophic lateral sclerosis

## bio.201410066 Supplementary Material

**Files in this Data Supplement:**

- Supplementary Material - Senthilkumar Deivasigamani et al. doi: 10.1242/bio.201410066
- Table S1 - **A list of the 103 modifiers (58 suppressors, 45 enhancers) of VAP function discovered in our genetic screen.** The modifiers are listed in alphabetical order of their Flybase symbol.
- Table S2 - **Modifiers identified in our screen that in earlier studies have been implicated in several neuronal disorders.** Known ALS loci identified in our screen are not a part of this list.
- Table S3 - **Conserved interactors of VAP found in other studies/organisms.**
