# Supplementary material for: A genetic screen identifies Tor as an interactor of VAPB in a Drosophila model of amyotrophic lateral sclerosis
Source: Biol Open. 2014 Oct 31;3(11):1127–38. doi: 10.1242/bio.201410066 (PMC4232771; doi:10.1242/bio.201410066)
Supplement: Supplementary Material [file supp_bio.201410066_bio.201410066-s1.pdf]

Supplementary Material
Senthilkumar Deivasigamani et al. doi: 10.1242/bio.201410066

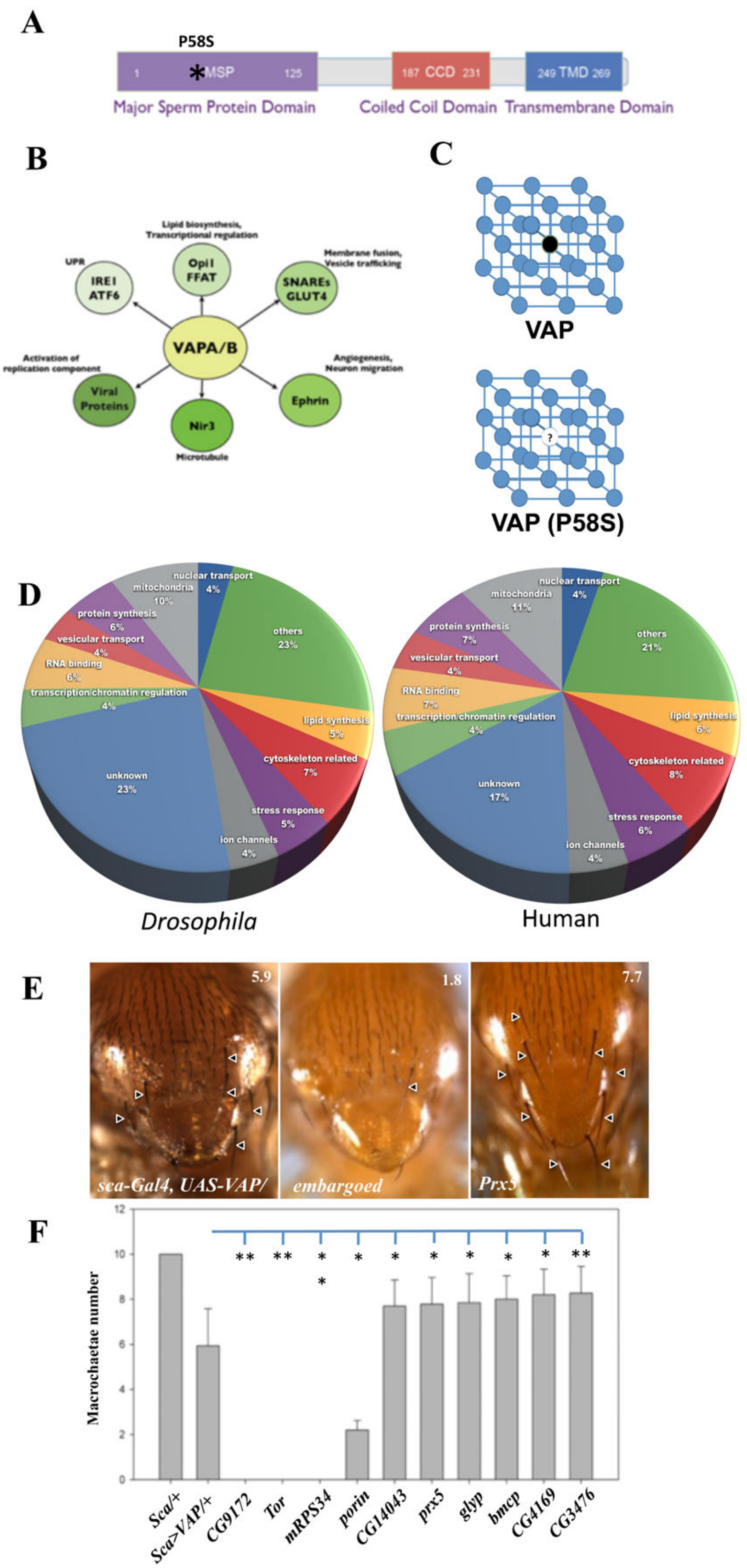

Fig. S1. VAP, VAP functions, VAP network and categorization of modifiers. (A) *Drosophila* VAP (dVAP) is a single pass trans-membrane protein (TMD) containing a coiled coil domain and an N-terminal major sperm protein (MSP) domain. A P56S mutation in the human ortholog of dVAP is linked to Amyotrophic Lateral sclerosis (ALS), a motor neuron disease. (B) VAP, with its cytoplasmic/ER localization, with the TMD inserted in the ER membrane interacts physically with a large number of proteins and has been demonstrated to have roles in diverse cellular function. (C) We hypothesize that a subset of genetic interactors, when discovered for VAP, may modify dVAP related phenotypes. (D) Graphical, GO representation for *Drosophila* 103 VAP interactors and also their human homologs. (E) Representative examples of thoracic macro chaetae for modifiers along with control, *sca-Gal4,UAS-VAP/+*. *Embargoed*, a nuclear pore protein is an enhancer of VAP function while *Prx5* involved in oxidative stress is a suppressor. (F) Average Macrochaetae number in ten genes involved in energy and mitochondrial metabolism. A full list of the 103 interactors can be found in supplementary material Table S1. * indicates a p-value<0.01 (but >0.001), while ** indicates a p-value of <0.001.

Biology Open

Biology Open (2014) 000, 1–12 doi:10.1242/bio.201410066

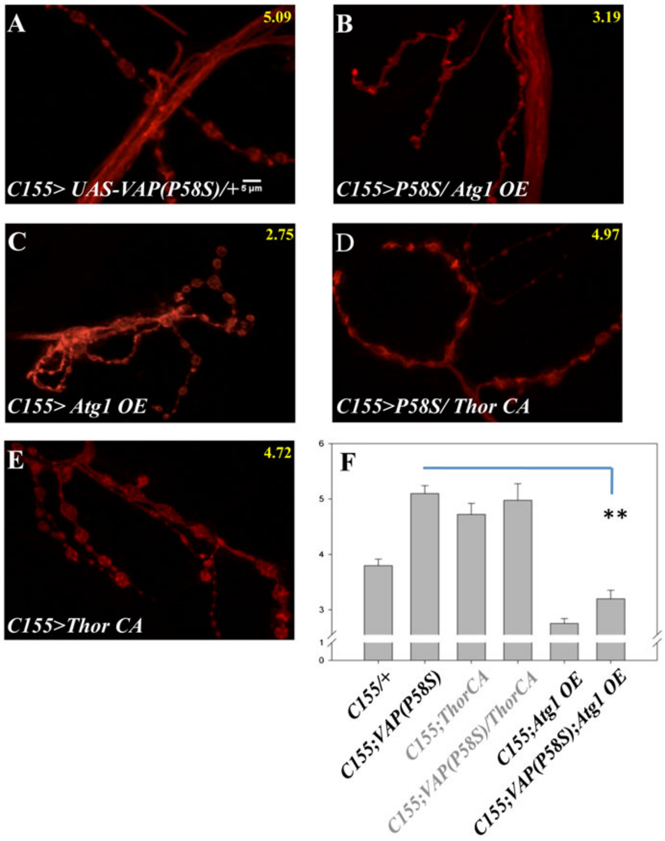

Fig. S2. TOR downstream components ATG1 and 4EBP1 (Thor) modulate the VAP(P58S) bouton phenotype. (A) VAP(P58S) over-expression using *C155-Gal4* leads to larger boutons at the NMJ. (B) Over-expression of *Atg1* in VAP(P58S) background reduces the bouton size, to levels lower than wild type. (C) Over-expression of *Atg1* alone using *C155-Gal4* resulted in reduced bouton size. (D) Over-expression of a constitutively active form 4EBP1 (Thor) does not recue the bouton size. (E) Over-expression of constitutively active 4EBP1 leads to increased bouton size. Scale bar: 5 µm. Average size of boutons from about 15 NMJs is displayed in yellow at the top right of each figure. (F) Quantitation of effect of TOR downstream effectors in VAP(P58S) mediated bouton size. Error bars represent SEM. * indicates a p-value<0.01 (but >0.001), while ** indicates a p-value of <0.001.

Biology Open

Biology Open (2014) 000, 1–12 doi:10.1242/bio.201410066

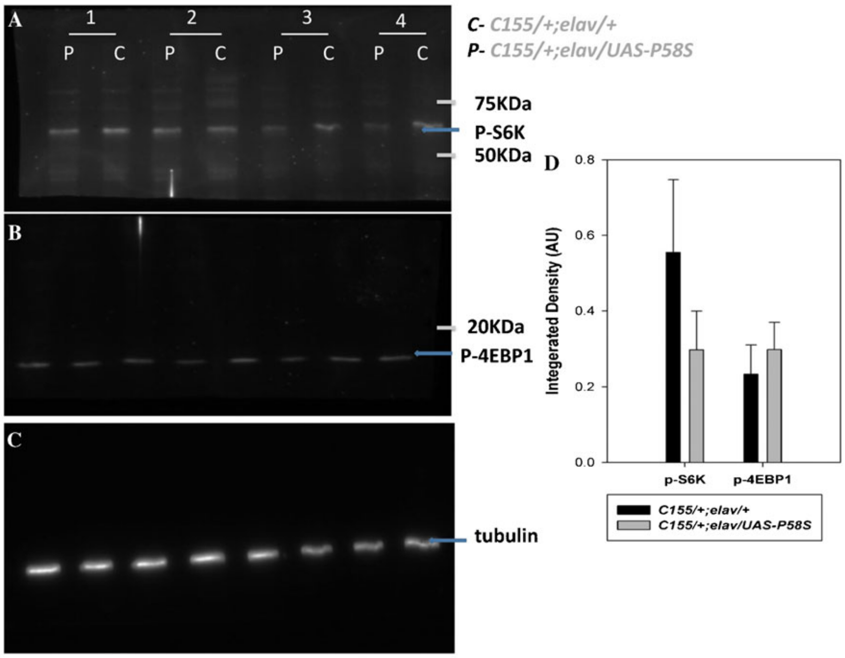

Fig. S3. Western blots of downstream effectors of TOR signaling. Western blots of larval brains from *C155/+,elav/+* (lanes marked as C) and *C155/+,elav/UAS-VAP(P58S)* (lanes marked as P). Phospho-S6K (A) and Phospho-4EBP1/Thor (B) levels do not change significantly ($p>0.01$), when normalized to tubulin (C). Quantitation of band intensity (D) is also displayed. The data includes four biological replicates (labeled 1, 2, 3 and 4).

Table S1-S3. See supplementary webpage.

Biology Open
