# Supplementary material for: A genetic screen identifies Tor as an interactor of VAPB in a Drosophila model of amyotrophic lateral sclerosis
Source: Biol Open. 2014 Oct 31;3(11):1127–38. doi: 10.1242/bio.201410066 (PMC4232771; doi:10.1242/bio.201410066)
Supplement: Supplementary Material [file supp_bio.201410066_Table_S1.docx]

**Supplementary Table 1**: A list of the 103 modifiers (58 Suppressors, 45 Enhancers) of VAP functions discovered in our genetic screen. The modifiers are listed in alphabetical order of their Flybase symbol.

| **Symbol** | **Name** | **Modifier class** | **Flybase Gene Number** | **Function** |
| --- | --- | --- | --- | --- |
| *Ars2* | *-* | Enhancer | FBgn0033062 | response to arsenic, |
|  |  |  |  | response to arsenic, catalytic step2 spliceosome |
|  |  |  |  | negative regulation of viral genome replication |
|  |  |  |  | conversion of ds siRNA to ss siRNA involved in RNA interference |
|  |  |  |  | gene silencing by miRNA |
|  |  |  |  | nuclear mRNA splicing, via spliceosome |
| *Bmcp* | *Bmcp* | Suppressor | FBgn0036199 | regulation of metabolic process,mitochondrial transmembrane transport |
| *Bx* | *Beadex* | Enhancer | FBgn0000242 | imaginal disc-derived leg segmentation |
|  |  |  |  | leg disc development |
|  |  |  |  | locomotor rhythm |
|  |  |  |  | response to cocaine |
|  |  |  |  | phagocytosis, engulfment |
|  |  |  |  | inter-male aggressive behavior |
| *Cdc6* | *Cdc6* | Enhancer | FBgn0035918 | pre-replicative complex assembly |
| *CG10089* | *-* | Suppressor | FBgn0036369 | protein dephosphorylation |
| *CG11560* | *-* | Suppressor | FBgn0036249 | - |
| *CG11811* | *-* | Suppressor | FBgn0036099 | purine nucleotide metabolic process |
| *CG12743* | *ovarian tumor* | Enhancer | FBgn0003023 | female germ-line sex determination, |
|  |  |  |  | germ cell development |
|  |  |  |  | germ cell development |
|  |  |  |  | germ-line cyst formation |
|  |  |  |  | germ-line sex determination |
|  |  |  |  | oogenesis |
|  |  |  |  | chromosome organization |
|  |  |  |  | positive regulation of DNA endoreduplication |
| *CG12822* | *-* | Suppressor | FBgn0033229 | - |
| *CG13048* | *-* | Enhancer | FBgn0036593 | - |
| *CG13063* | *-* | Suppressor | FBgn0036601 | - |
| *CG13075* | *-* | Suppressor | FBgn0036563 | chitin metabolic process |
| *CG13296* | *-* | Enhancer | FBgn0035687 | nucleic acid binding |
| *CG13387* | *embargoed* | Enhancer | FBgn0020497 | protein export from nucleus, |
|  |  |  |  | multicellular organismal development |
|  |  |  |  | protein export from nucleus |
|  |  |  |  | protein transport |
|  |  |  |  | protein export from nucleus |
|  |  |  |  | nuclear export |
|  |  |  |  | centriole replication |
| *CG13994* | *-* | Enhancer | FBgn0031772 | - |
| *CG14043* | *-* | Suppressor | FBgn0031659 | - |
| *CG14125* | *-* | Suppressor | FBgn0036232 | chitin metabolic process |
| *CG14222* |  | Enhancer | FBgn0031043 | metabolic process |
| *CG14326* | *-* | Suppressor | FBgn0038528 | - |
| *CG14435* | *smallminded* | Enhancer | FBgn0029911 | - |
| *CG14606* | *-* | Suppressor | FBgn0037485 | phagocytosis, engulfment, hexose transmembrane transport |
| *CG14837* | *-* | Suppressor | FBgn0035797 | - |
| *CG15160* | *-* | Enhancer | FBgn0032688 | - |
| *CG17327* | *-* | Suppressor | FBgn0038107 | translation |
| *CG17760* | *-* | Suppressor | FBgn0033756 | G-protein coupled receptor protein signaling pathway, protein ADP-ribosylation |
| *CG17985* | *-* | Suppressor | FBgn0033199 | cell wall macromolecule catabolic process |
| *CG18110* | *-* | Suppressor | FBgn0039677 | sodium ion transport |
| *CG30043* | *-* | Suppressor | FBgn0050043 | proteolysis |
| *CG30060* | *-* | Suppressor | FBgn0050060 | - |
| *CG30161* | *-* | Enhancer | FBgn0050161 | neurogenesis |
| *CG31702* | *-* | Enhancer | FBgn0051702 | transcription initiation, DNA-dependent |
| *CG31769* | *-* | Suppressor | FBgn0051769 | biological_process |
| *CG32437* | *-* | Suppressor | FBgn0052437 | - |
| *CG32685* | *-* | Suppressor | FBgn0052685 | - |
| *CG32703* | *-* | Suppressor | FBgn0052703 | protein phosphorylation |
| *CG33090* | *-* | Suppressor | FBgn0028916 | bile acid metabolic process, |
|  |  |  |  | O-glycoside catabolic process |
|  |  |  |  | glucosylceramide catabolic process |
| *CG34113* | *-* | Enhancer | FBgn0083949 | - |
| *CG3476* | *-* | Suppressor | FBgn0031881 | acyl carnitine transport, |
|  |  |  |  | carnitine transport |
|  |  |  |  | mitochondrial transport |
|  |  |  |  | transmembrane transport |
| *CG3500* | *-* | Enhancer | FBgn0034849 | - |
| *CG3884* | *-* | Enhancer | FBgn0033786 | - |
| *CG3975* | *-* | Suppressor | FBgn0027559 | DNA replication |
| *CG4169* | *-* | Suppressor | FBgn0250814 | mitochondrial electron transport, ubiquinol to cytochrome c, |
|  |  |  |  | proteolysis |
|  |  |  |  | lipid particle associated |
|  |  |  |  | microtubule associated complex |
| *CG42255* | *-* | Suppressor | FBgn0259140 | Microtubule associated complex, calcium binding, metalloendopeptidase |
| *CG4627* | *-* | Suppressor | FBgn0033808 | - |
| *CG4646* | *-* | Suppressor | FBgn0033810 | biological_process |
| *CG5325* | *-* | Suppressor | FBgn0032407 | nervous system development , peroxisome |
| *CG5733* | *Nucleoporin 75* | Enhancer | FBgn0034310 | SMAD protein nuclear translocation,NLS-bearing substrate import into nucleus |
| *CG5953* | *-* | Suppressor | FBgn0032587 | - |
| *CG6048* | *-* | Suppressor | FBgn0029827 | proteolysis |
| *CG6220* | *Enhancer of Polycomb* | Enhancer | FBgn0033865 | - |
| *CG6345* | *-* | Suppressor | FBgn0037816 | regulation of cyclin-dependent protein kinase activity,tRNA modification |
| *CG6502* | *Enhancer of zeste* | Enhancer | FBgn0000629 | histone methylation, |
|  |  |  |  | chromatin silencing |
|  |  |  |  | histone methylation |
|  |  |  |  | syncytial blastoderm mitotic cell cycle |
|  |  |  |  | axon guidance |
|  |  |  |  | muscle organ development |
|  |  |  |  | dendrite morphogenesis |
|  |  |  |  | neurogenesis |
|  |  |  |  | histone H3-K27 methylation |
|  |  |  |  | histone H3-K27 methylation |
|  |  |  |  | histone H3-K9 methylation |
|  |  |  |  | histone methylation |
|  |  |  |  | cuticle hydrocarbon biosynthetic process |
| *CG6674* | *-* | Enhancer | FBgn0036063 | - |
| *CG6885* | *-* | Suppressor | FBgn0036810 | - |
| *CG6950* | *-* | Suppressor | FBgn0037955 | biosynthetic process |
| *CG7306* | *obstructor-F* | Enhancer | FBgn0036947 | chitin metabolic process |
| *CG7564* | *-* | Enhancer | FBgn0036734 | nuclear mRNA splicing, via spliceosome |
| *CG7564 (Alsin2)* | *-* | Suppressor | FBgn0037116 | Rab guanyl-nucleotide exchange factor activity,Regulator of chromosome condensation, RCC1; Vacuolar sorting protein 9 |
| *CG7776* | *-* | Enhancer | FBgn0000581 | chromatin organization |
| *CG8219* | *-* | Enhancer | FBgn0035693 | protein import into nucleus, nuclear pore |
| *CG8571* |  | Enhancer | FBgn0016983 | - |
| *CG8863* | *DnaJ-like-2* | Enhancer | FBgn0038145 | protein folding, response to heat |
| *CG9172* | *-* | Enhancer | FBgn0030718 | mitochondrial electron transport, NADH to ubiquinone, |
|  |  |  |  | determination of adult lifespan |
|  |  |  |  | mitochondrial respiratory chain complex I assembly |
|  |  |  |  | response to reactive oxygen species |
| *CG9391* |  | Enhancer | FBgn0037063 | dephosphorylation |
| *CG9638* | *Ada2b* | Enhancer | FBgn0037555 | positive regulation of histone acetylation, |
|  |  |  |  | regulation of transcription , SAGA complex, polytene chromosome |
|  |  |  |  | chromatin remodeling |
|  |  |  |  | cell proliferation |
|  |  |  |  | histone H3 acetylation |
|  |  |  |  | eye pigment metabolic process |
| *CG9953* | *-* | Enhancer | FBgn0035726 | proteolysis |
| *Cyp4ac1* | *Cyp4ac1* | Suppressor | FBgn0031693 | hormone metabolic process, insecticide catabolic process |
| *Cyp6a19* | *Cyp6a19* | Suppressor | FBgn0033979 | oxidation-reduction process |
| *Cyp6a2* | *Cytochrome P450-6a2* | Suppressor | FBgn0000473 | response to insecticide, response to caffeine, oxidation-reduction process |
| *Cyp9b2* | *Cytochrome P450-9b2* | Suppressor | FBgn0015039 | microsome |
| *dila* | *dilatory* | Suppressor | FBgn0033447 | adult locomotory behavior, cilium axoneme assembly, spermatogenesis, peripheral nervous system neuron development |
| *dyn-p25* | *dynactin-subunit-p25* | Enhancer | FBgn0040228 | microtubule-based movement |
| *ear* | *ENL/AF9-related* | Suppressor | FBgn0026441 | transcription initiation from RNA polymerase II promoter,regulation of transcription, DNA-dependent |
| *Ef1beta* | *Elongation factor 1 beta* | Enhancer | FBgn0028737 | translational elongation |
| *eIF2B-delta* | *eIF2B-delta* | Enhancer | FBgn0034858 | translational initiation, neurogenesis |
| *epsilonCOP* | *epsilonCOP* | Suppressor | FBgn0027496 | retrograde vesicle-mediated transport, Golgi to ER |
| *GlyP* | *Glycogen phosphorylase* | Suppressor | FBgn0004507 | glycogen catabolic process, flight |
| *Hsp83* | *Heat shock protein 83* | Suppressor | FBgn0001233 | Spermatogenesis, |
| *icln* | *Icln* | Enhancer | FBgn0029079 | cell volume homeostasis , chloride transport , volume sensitive anion channel |
| *Irp-1B* | *Iron regulatory protein 1B* | Suppressor | FBgn0024957 | regulation of translational initiation by iron |
| *Karybeta3* | *Karyopherin beta 3* | Enhancer | FBgn0087013 | protein import into nucleus |
| *l(1)G0222* | *lethal (1) G0222* | Suppressor | FBgn0028343 | - |
| *l(2)35Cc* | *lethal (2) 35Cc* | Enhancer | FBgn0259982 | unfolded protein binding,protein folding |
| *mRpS34* | *mitochondrial ribosomal protein S34* | Enhancer | FBgn0260460 | mitochondrial small ribosomal subunit |
| *mys* | *myospheroid* | Enhancer | FBgn0004657 | central nervous system development,cell migration |
|  |  |  |  | midgut development |
|  |  |  |  | muscle attachment |
|  |  |  |  | calcium-dependent cell-cell adhesion |
|  |  |  |  | calcium-dependent cell-matrix adhesion |
|  |  |  |  | cell-matrix adhesion |
|  |  |  |  | heterophilic cell-cell adhesion |
|  |  |  |  | apposition of dorsal and ventral imaginal disc-derived wing surfaces |
|  |  |  |  | cell adhesion |
|  |  |  |  | regulation of cell shape |
|  |  |  |  | axon guidance |
|  |  |  |  | dorsal closure |
|  |  |  |  | epithelial cell migration, open tracheal system |
|  |  |  |  | tracheal outgrowth, open tracheal system |
|  |  |  |  | maintenance of epithelial integrity, open tracheal system |
|  |  |  |  | hemocyte migration |
|  |  |  |  | substrate-dependent cell migration, cell extension, |
|  |  |  |  | determination of adult lifespan |
|  |  |  |  | germ-line stem cell maintenance |
|  |  |  |  | sensory perception of smell |
|  |  |  |  | imaginal disc-derived male genitalia morphogenesis |
|  |  |  |  | cell-substrate adhesion |
|  |  |  |  | integrin-mediated signaling pathway |
|  |  |  |  | cell-matrix adhesion |
| *NaPi-T* | *Na[+]-dependent inorganic phosphate cotransporter* | Suppressor | FBgn0016684 | phosphate transport |
| *Nlp* | *Nucleoplasmin* | Enhancer | FBgn0016685 | nucleosome positioning |
| *Osi20* | *Osiris 20* | Suppressor | FBgn0037430 | biological_process unknown |
| *pallidin* | *pallidin* | Enhancer | FBgn0036192 | - |
| *porin* | *porin* | Enhancer | FBgn0004363 | mitochondrial transport , |
|  |  |  |  | mitochondrial transport |
|  |  |  |  | ion transport |
|  |  |  |  | phototransduction |
|  |  |  |  | sperm individualization |
|  |  |  |  | mitochondrion organization |
|  |  |  |  | sperm mitochondrion organization |
|  |  |  |  | transmembrane transport |
|  |  |  |  | Enhancer regulation of anion transport |
| *PrBP* | *Prenyl-binding protein* | Enhancer | FBgn0032059 | mushroom body development,visual perception , 3',5'-cyclic-GMP phosphodiesterase activity |
| *Prx5* | *Peroxiredoxin 5* | Suppressor | FBgn0038570 | response to oxidative stress, peroxisome, mitochondrion, cell redox homeostasis |
| *snama* | *something that sticks like glue* | Suppressor | FBgn0086129 | - |
| *SOD1* | *Superoxide dismutase* | Suppressor | FBgn0003462 | Superoxide dismutase (Cu/Zn) / chaperones; Superoxide dismutase, copper/zinc binding domain; Superoxide dismutase, copper/zinc, binding site, response to oxidative stress |
| *ssh* | *slingshot* | Enhancer | FBgn0029157 | protein dephosphorylation, |
|  |  |  |  | actin cytoskeleton organization |
|  |  |  |  | regulation of actin polymerization or depolymerization |
|  |  |  |  | mushroom body development |
|  |  |  |  | regulation of axonogenesis |
|  |  |  |  | regulation of lamellipodium assembly |
|  |  |  |  | mitotic cell cycle |
|  |  |  |  | protein dephosphorylation |
| *Su(P)* | *Suppressor of ref(2)P sterility* | Suppressor | FBgn0004465 | cell redox homeostasis, protein disulfide oxidoreductase |
| *TBPH* | *TBPH* | Suppressor | FBgn0025790 | neuromuscular junction development,adult locomotory behavior, RNA metabolism |
| *tectonic* | *tectonic* | Suppressor | FBgn0261697 | regulation of smoothened signaling pathway |
| *tmod* | *tropomodulin* | Suppressor | FBgn0082582 | cytoskeleton organization |
| *Tor* | *Target of rapamycin* | Enhancer | FBgn0021796 | Growth, |
|  |  |  |  | response to nutrient |
|  |  |  |  | phosphorylation |
|  |  |  |  | detection of nutrient |
|  |  |  |  | positive regulation of cell size |
|  |  |  |  | positive regulation of organ growth |
|  |  |  |  | positive regulation of cell growth |
|  |  |  |  | positive regulation of multicellular organism growth |
|  |  |  |  | determination of adult lifespan |
|  |  |  |  | autophagy |
|  |  |  |  | multicellular organism growth |
|  |  |  |  | gonad development |
|  |  |  |  | positive regulation of cell growth |
|  |  |  |  | endocytic recycling |
|  |  |  |  | regulation of cell growth |
|  |  |  |  | positive regulation of cell size |
|  |  |  |  | positive regulation of ribosome biogenesis |
|  |  |  |  | response to DNA damage stimulus |
|  |  |  |  | dendrite morphogenesis |
| *torp4a* | *torp4a* | Suppressor | FBgn0025615 | protein folding, Endoplasmic reticulum lumen |
| *Tsp2A* | *Tetraspanin 2A* | Enhancer | FBgn0024361 | integral to membrane |
| *Use1* | *Use1* | Suppressor | FBgn0035965 | Golgi organization, neurogenesis, protein secretion |
| *Zif* | *Zinc-finger protein* | Enhancer | FBgn0037446 | establishment or maintenance of neuroblast polarity, neuroblast proliferation |
| *zye* | *zye* | Suppressor | FBgn0036985 | regulation of embryonic cell shape, filamentous actin |
|  |  |  |  | cell-matrix adhesion |
|  |  |  |  | actin filament organization |
