# Supplementary material for: A genetic screen identifies Tor as an interactor of VAPB in a Drosophila model of amyotrophic lateral sclerosis
Source: Biol Open. 2014 Oct 31;3(11):1127–38. doi: 10.1242/bio.201410066 (PMC4232771; doi:10.1242/bio.201410066)
Supplement: Supplementary Material [file supp_bio.201410066_Table_S2.doc]

**Supplementary Table 2:** 22 of the 103 modifiers identified in our screen have been implicated in several neuronal disorders. Known ALS loci (*TBPH, alsin2, sod1*) identified in our screen are not a part of this list.

| **FLY GENE** | **MAMMALIAN ORTHOLOG** | **MOLECULAR FUNCTION**  **(FLIES AND HUMANS)** | **ASSOCIATED NERVOUS SYSTEM DISORDER** | **REFERENCE/ SOURCE** |
| --- | --- | --- | --- | --- |
| *Karyopherin beta-3* | *IPO5* | Nuclear export/import | Huntington’s Disease | Zhang et. al. (2010) |
| *Target of Rapamycin* | *MTOR* | Nutrient sensing, energy homeostasis, protein synthesis | Huntington’s Disease | Ravikumar et. al.(2004) |
| *Ada2b* | *TADA2B* | Chromatin remodelling | Huntington’s Disease | Zhang et. al. (2010) |
| *Ars2* | *SRRT* | mRNA binding | Huntington’s Disease | Zhang et. al. (2010) |
| *CG6950* | *CCBL2* | Kynurenine-Oxologlutarate Transaminase | Parkinson’s Disease | Hartai et. al. (2005) |
| *CG9172* | *NDUFS7* | NADH dehydrogenase I (ubiquinone) Fe-s | Huntington’s Disease, Leigh syndrome due to cytochrome c oxidase deficiency | Kaltenbach et. al. (2007) |
| *Hsp83* | *HSP90AA1* | Chaperone | Huntington’s Disease, Alzheimer’s Disease | Zhang et. al. (2010), van Ham et. al. (2008) |
| *CG8219/Transportin* | *TNPO1* | Nuclear export/import | Amyotrophic Lateral Sclerosis/FUS | Dormann et. al. (2010) |
| *icln* | *CLNS1A* | Chloride channel, Methylosome | Spinal Muscular Atrophy | Dormann et. al. (2010) |
| *E(Pc)* | *EPC2* | Transcription factor | Alzheimer's disease (biomarkers) | DIOPT-DIST/OMIM |
| *CG5325* | *PEX19* | Peroxisome biogenesis | Peroxisome biogenesis disorder 12A | DIOPT-DIST/OMIM |
| *CG14043* | *KIAA1279* | Unknown | Goldberg-Shprintzen megacolon syndrome | DIOPT-DIST/OMIM |
| *CG3476* | *SLC25A20* | Acyl carnitine transporter | Carnitine-acylcarnitine translocase deficiency | DIOPT-DIST/OMIM |
| *CG9391* | *IMPA2* | Inositol metabolism | Stroke | DIOPT-DIST/OMIM |
| *eIF2B-delta* | *EIF2B4* | Translation initiation | Leukoencephalopathy with vanishing white matter | DIOPT-DIST/OMIM |
| *smid* | *NVL* | Valosin containing protein | Taupathy | Ambegaonkar et. al. (2011) |
| *dyn-p25* | *DCTN5* | microtubule-based movement | Bipolar Disorder | DIOPT-DIST/OMIM |
| *ear* | *AP5Z1* | Transcription factor | Spastic Paraplegia | DIOPT-DIST/OMIM |
| *Irp-1B* | *ACO2* | Translation and metabolism | Infantile cerebellar-retinal degeneration | DIOPT-DIST/OMIM |
| *NaPi-T* | *SLC17A5* | Ion channel | Salla Disease | DIOPT-DIST/OMIM |
| *tectonic* | *BD92/ TCTN3* | Smoothened signaling | Joubert syndrome, Meckel Syndrome | DIOPT-DIST/OMIM |
| *ZAP/ CG32685* | *AP5Z1* | Unknown | Spastic Paraplegia | DIOPT-DIST/OMIM |
