# Supplementary material for: A genetic screen identifies Tor as an interactor of VAPB in a Drosophila model of amyotrophic lateral sclerosis
Source: Biol Open. 2014 Oct 31;3(11):1127–38. doi: 10.1242/bio.201410066 (PMC4232771; doi:10.1242/bio.201410066)
Supplement: Supplementary Material [file supp_bio.201410066_Table_S3.docx]

**Supplementary Table 3**: Conserved interactors of *VAP* found in other studies/organisms.

| **Gene** | **Function** | **Conserved in** |
| --- | --- | --- |
| *ssh* | cytoskeleton organisation | Human |
| *tmod* | cytoskeleton organisation | Human |
| *GlyP* | glycogen metabolism | Human |
| *CG17760* | GPCR | Human |
| *Cyp4ac1* | hormone metabolism | Human |
| *Bx* | locomotor rhythm | Human |
| *Cyp6a19* | monoxygenase | Human |
| *Zif* | neuroblast polarity | Human |
| *Pex19* | Peroxisome biogenesis | Yeast |
| *Cyp9b2* | smoothened signaling | Human |
| *Ef1beta* | translation elongation | Human |
| *Use1* | Ubiqutin ligase | Yeast |
| *CG13296* | unknown | Human |
| *smid* | unknown | Human |
| *CG32703* | unknown | Human |
| *CG34113* | unknown | Human |
| *Tsp2A* | unknown | Human |
| *Nlp* | nuclear chaperone | Human |
| *TBPH* | RNA splicing | Human |
